# Supplementary material for: Visualization of the Association of Dimeric Protein Complexes on Specific Enhancers in the Salivary Gland Nuclei of Drosophila Larva
Source: Cells. 2024 Apr 1;13(7):613. doi: 10.3390/cells13070613 (PMC11012150; doi:10.3390/cells13070613)
Supplement: Supplementary file 1 [file cells-13-00613-s001.zip › cells-2908361-supplementary.pdf]

A

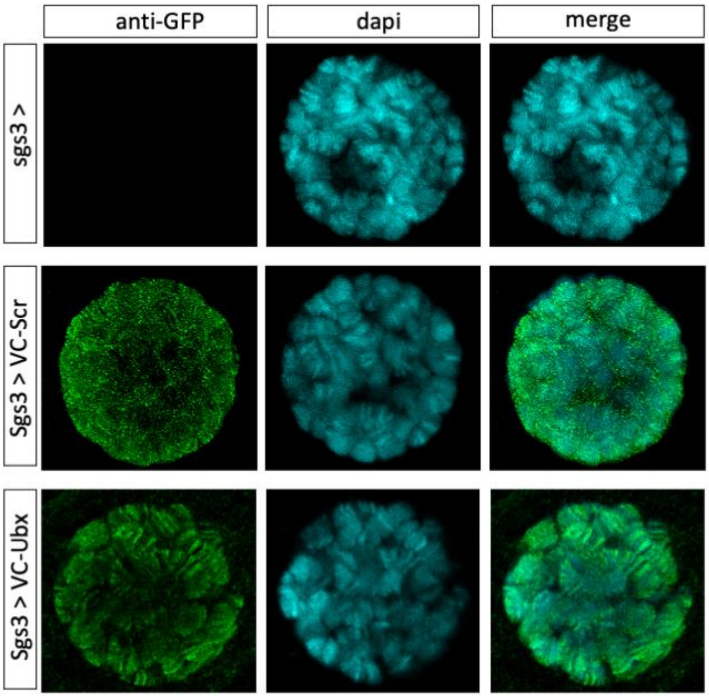

B

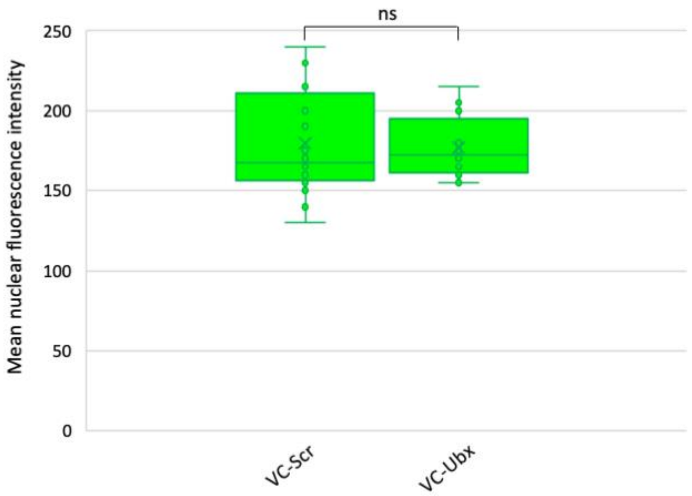

Supplementary Figure 1. VC-Scr and VC-Ubx expression in salivary gland nuclei with *the sgs3-Gal4* driver. A. Illustrative deconvoluted confocal acquisitions of the immunostaining against VC-Hox constructs revealed with an anti-GFP antibody recognizing the VC fragment (green). Dapi (cyan) stains for polytene chromosomes. Acquisitions were taken with a 40x objective. **B.** Box plot representation of the quantification of the GFP fluorescent immunostaining in salivary gland nuclei (established from two biological replicates). ns: non-significant. See also material and methods.

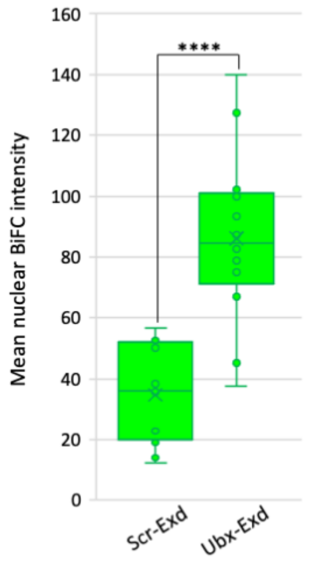

Supplementary Figure 2. Statistical quantification of BiFC in whole salivary gland nuclei. Box plot representation of the mean fluorescence intensity resulting from BiFC between Scr/Exd or Ubx/Exd in salivary gland nuclei. Measurements are from non-deconvoluted acquisitions with a 63x objective. BiFC resulting from Ubx/Exd complexes is significantly higher than BiFC resulting from Scr/Exd complexes. pvalues ****≤0,00001 is calculated from the quantification of three independent biological replicates. See also material and methods.

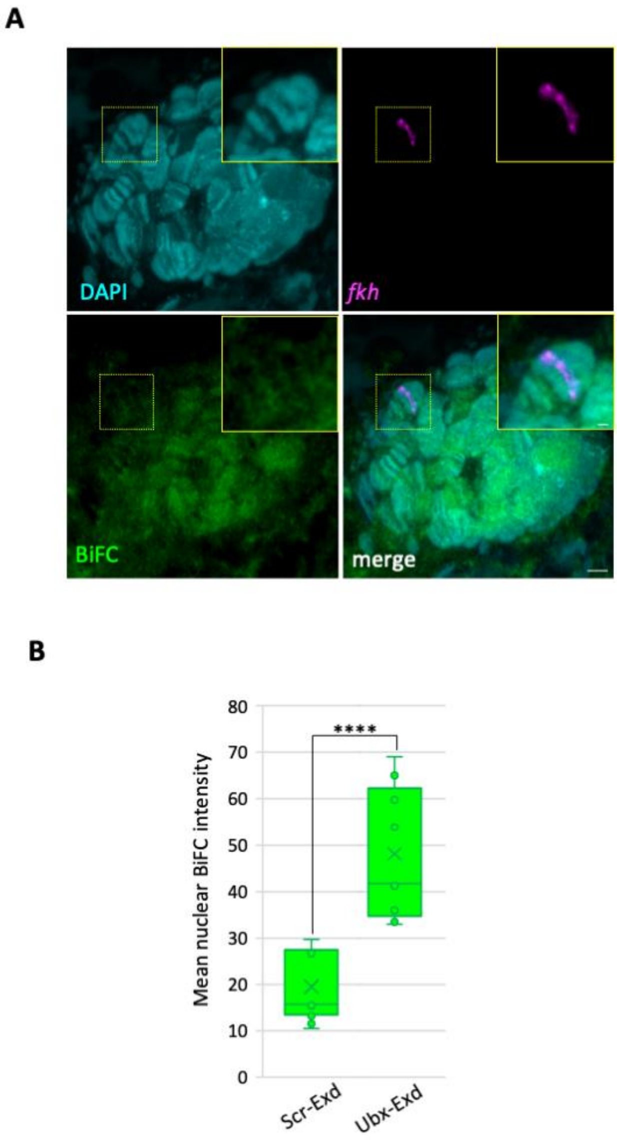

Supplementary Figure 3. BiFC under FISH experimental conditions. A. Confocal acquisition without lightning deconvolution of BiFC signals resulting from Ubx/Exd association. The illustrative nucleus is the same as in the Fig. 2. Very few nuclei (one or two/ experiment) show BiFC signals (green) that are not-well defined (both at the intensity and resolution levels). Enlargement depicts the *fkh* genomic locus stained by FISH (magenta). **B.** Box plot representation of the mean BiFC intensity resulting from VC-Scr/VN-Exd or VC-Ubx/VN-Exd complex assembly, as indicated. The fluorescence intensity measured for VC-Scr/VN-Exd corresponds to the background. Quantifications are from two biological replicates and from non-deconvoluted acquisitions. Scale bars=5µm (upper panels) or 1µm (enlargements).

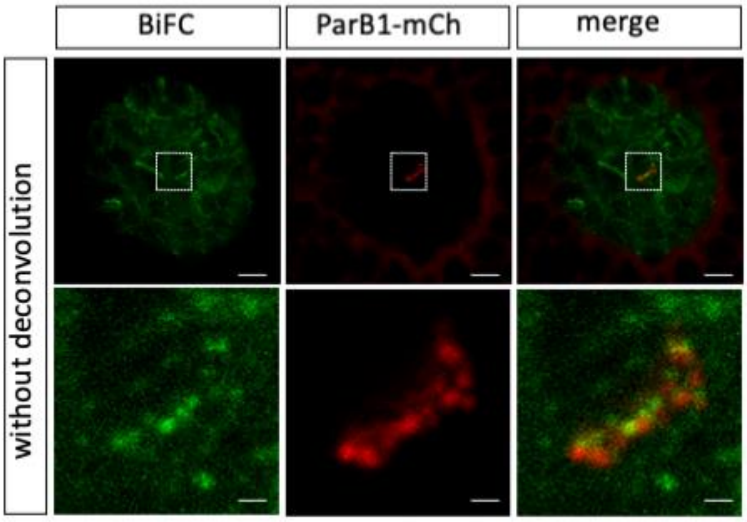

Supplementary Figure 4. Confocal acquisition without deconvolution of VC-Scr/VN-Exd BiFC in a *INT1-fkh250*/ParB1-mCherry positive salivary gland nucleus. The nucleus is the same as the one shown in Fig. 3A′. Scale bars=5µm (upper panels) or 1µm (enlargements).

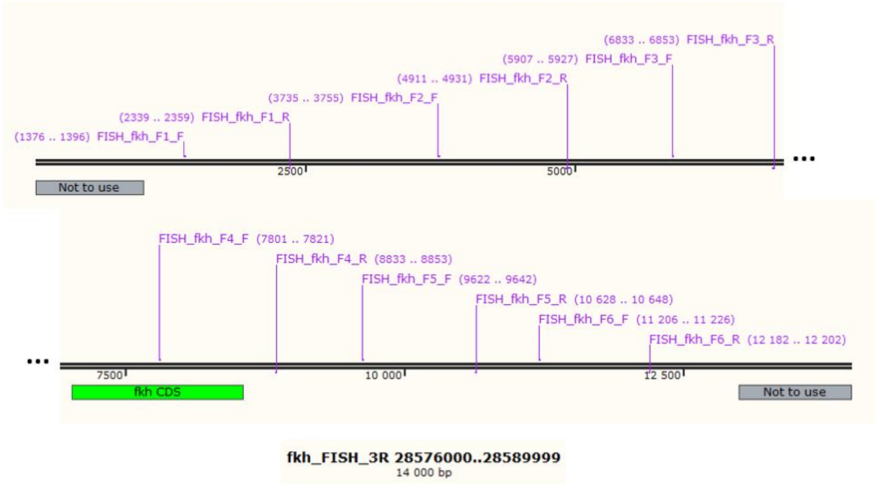

Supplementary Figure 5. Scheme of the different DNA probes designed around the *forkhead (fkh)* gene for doing DNA FISH in salivary gland nuclei. Adapted from Flybase (https://flybase.org/).

A

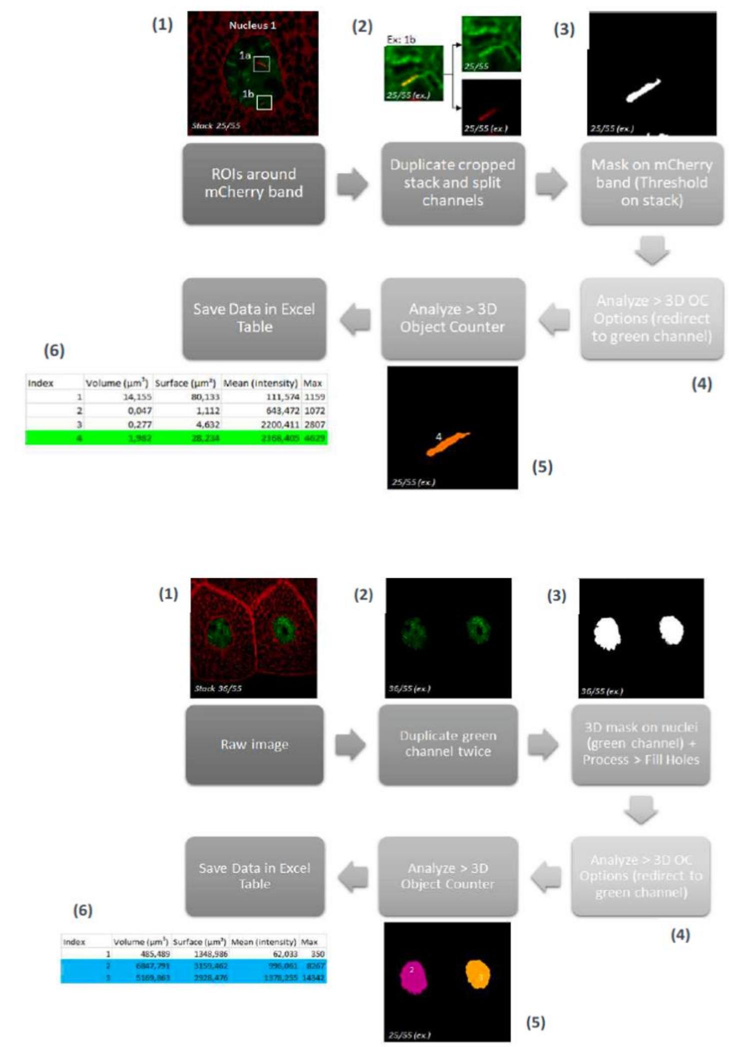

Supplementary Figure 6. Pipelines for quantifying BiFC/immunostaining signals on fkh enhancers or in the whole nucleus. A. Quantification of BiFC signals on *fkh* enhancers. On the raw image, draw a region of interest (ROI) around one enhancer of the first nucleus (step 1). Duplicate crop and separate channels (step 2). Choose the image with the most intense red band for thresholding (to avoid overestimating band width). Create the binary mask (step 3) that will be used to apply the "3D Object Counter" plug-in. In the Plugin options, check the "Volume", "Surface", "Min", "Max" and "Mean" parameters and redirect measurements to the original green channel (step 4). Launch the "3D Object Counter" plug-in with a minimum size of 10 and a default maximum size ("size filter") (step 5). Data are extracted in 3D from the entire enhancer and saved as an Excel table (step 6). Intensities measured in the mask correspond to those found in the BiFC image (redirection to the green channel). This operation is repeated for the different nuclei in the image. **B.** Quantification of BiFC signals in the whole nucleus. The same principle is applied to the whole nucleus. No ROI is required for this process. The 3D threshold on the green channel enables the "3D Object Counter" plug-in to be applied, with the same parameters as above, except for the default minimum size = 100,000 and maximum size ("size filter") (step 5). Finally, the extracted data are used to compare the different conditions. To determine the enrichment of the BiFC signal on the enhancer, the mean maximum value of the gray level maxima in one enhancer is related to the maximum gray level value in the corresponding nucleus. This ratio is applied to each enhancer in the different nuclei and in all conditions.
